# Biogeochemical feedbacks to ocean acidification in a cohesive photosynthetic sediment

**DOI:** 10.1038/s41598-021-02314-y

**Published:** 2021-11-24

**Authors:** Kay Vopel, Alexis Marshall, Shelly Brandt, Adam Hartland, Charles K. Lee, S. Craig Cary, Conrad A. Pilditch

**Affiliations:** 1 School of Science, Auckland University of Technology, Private Bag 92006, Auckland, New Zealand; 2 School of Science, University of Waikato, Private Bag 3105, Hamilton, New Zealand; 3 Environmental Research Institute, School of Science, University of Waikato, Private Bag 3105, Hamilton, New Zealand

**Keywords:** Biogeochemistry, Climate-change ecology, Microbial ecology

## Abstract

Ecosystem feedbacks in response to ocean acidification can amplify or diminish diel pH oscillations in productive coastal waters. Benthic microalgae generate such oscillations in sediment porewater and here we ask how CO_2_ enrichment (acidification) of the overlying seawater alters these in the absence and presence of biogenic calcite. We placed a 1-mm layer of ground oyster shells, mimicking the arrival of dead calcifying biota (+Calcite), or sand (Control) onto intact silt sediment cores, and then gradually increased the *p*CO_2_ in the seawater above half of +Calcite and Control cores from 472 to 1216 μatm (pH 8.0 to 7.6, CO_2_:HCO_3_^−^ from 4.8 to 9.6 × 10^−4^). Porewater [O_2_] and [H^+^] microprofiles measured 16 d later showed that this enrichment had decreased the O_2_ penetration depth (O_2_-pd) in +Calcite and Control, indicating a metabolic response. In CO_2_-enriched seawater: (1) sediment biogeochemical processes respectively added and removed more H^+^ to and from the sediment porewater in darkness and light, than in ambient seawater *increasing* the amplitude of the diel porewater [H^+^] oscillations, and (2) in darkness, calcite dissolution in +Calcite sediment decreased the porewater [H^+^] below that in overlying seawater, reversing the sediment–seawater H^+^ flux and *decreasing* the amplitude of diel [H^+^] oscillations. This dissolution did not, however, counter the negative effect of CO_2_ enrichment on O_2_-pd. We now hypothesise that feedback to CO_2_ enrichment—an increase in the microbial reoxidation of reduced solutes with O_2_—decreased the sediment O_2_-pd and contributed to the enhanced porewater acidification.

## Introduction

Dissolution of anthropogenic CO_2_ in the ocean alters the speciation of dissolved inorganic carbon (DIC), increasing seawater [H^+^] while decreasing its carbonate saturation state (Ω) and buffering capacity^1–3^. A reduction in the buffering capacity implies that the future ocean will be more sensitive to CO_2_ addition than is presently the case and we can expect the current trends of increasing average seawater [H^+^]^4–6^ to be accompanied by an increase in the peak-to-peak amplitude of diel and seasonal variations in seawater [H^+^]^7^. If we now consider the possibility that biota conditioned to current diel and seasonal variations may respond to changes in the magnitude of these variations, independent of changes in the mean seawater carbonate chemistry, then knowledge of the effects of ocean acidification on these variations becomes important.

In productive coastal environments, metabolic (e.g., photosynthesis and respiration) and geochemical (e.g., precipitation and dissolution) processes have a relatively large influence on the seawater carbonate chemistry^8,9^. Feedbacks from these processes can enhance or diminish trends in both the mean seawater carbonate chemistry and its diel and seasonal variations. The magnitude of this effect depends on the metabolic and geochemical processes involved and the flushing of these environments with the open ocean, which determines the seawater residence time. For example, coastal eutrophication can amplify the amplitude of seawater [H^+^] variations by increasing primary production, which in turn tends to increase respiration^10–12^. Modelling a reef flat ecosystem, Jury et al.^13^ predicted that increasing seawater residence time and intensity of ecosystem feedbacks can increase diel [H^+^] variations by a factor of up to 2.5. In other systems, limitations in *vertical* solute exchange can also serve to enhance ecosystem feedbacks: Hagens et al.^14^, for example, found differences in the magnitude of seasonal [H^+^] fluctuations between the surface and bottom layer of a seasonally stratified coastal lake and attributed these differences to a reduction in the acid–base buffering capacity of the CO_2_-enriched bottom water in the summer period.

Here, we examine the effects of ecosystem feedbacks on diel variations in [H^+^] at the sediment–seawater boundary of photosynthetic silt—a cohesive sediment in which solute transport is dominated by molecular diffusion rather than porewater advection. The surface layer of this sediment, which separates the free-flowing seawater from the deeper anoxic layer, hosts a network of microbial mineralisation and solute reoxidation processes that operates at millimetre scales. Because solute transport is limited by molecular diffusion, this network creates steep, measurable gradients and, if photosynthesis is involved, strong diel oscillations in porewater [H^+^] and [O_2_]. We argue that feedbacks in response to ocean acidification occurring at this boundary matter for ecosystem-scale processes such as primary production, nutrient cycling, and the recruitment and dispersal of benthic fauna. This is because the environmental conditions at the sediment–seawater interface affect the cycling of carbon, nitrogen (including denitrification) and phosphorus and associated sediment–seawater solute exchanges, larval settlement, faunal emergence, and the behaviour of bioturbating species^15–18^.

Previously we found that experimental CO_2_ enrichment (acidification) of seawater overlying subtidal silt sediment shifted porewater [H^+^] profiles measured in light and darkness towards higher concentrations and that this shift extended to the depth of the anoxic sediment^19^. We now ask how such enrichment alters the diel oscillations in porewater [H^+^] and [O_2_] and if the dissolution of biogenic calcite, arriving at the sediment surface in form of dead calcifying biota, can dampen the amplitude of these oscillations. Besides porewater buffering, dissolution of calcite at the sediment surface may also have implications for recruitment of calcifying fauna^20–23^. In our design, we included measurements of porewater [O_2_] as a proxy of microbial metabolic activity to assess possible metabolic feedbacks to experimental enrichment of seawater with CO_2_.

**Figure 1:**
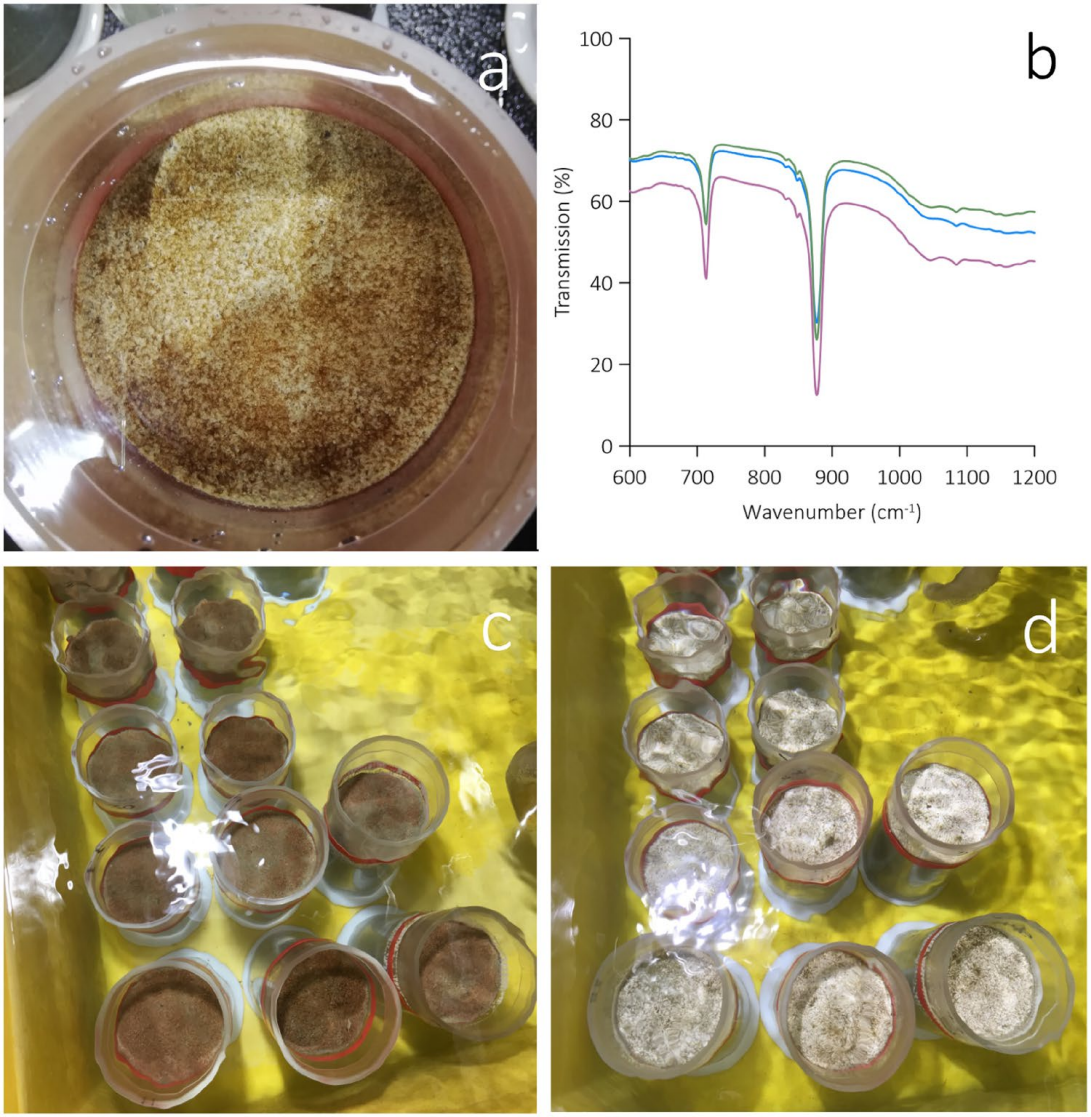
Photographs showing surfaces of intact cores of the subtidal silt sediment submerged in natural, recirculating seawater. The dark areas at the surface of the cores in (**a**) indicate the presence of pinnate diatoms. The three replicate Fourier Transform Infrared (FTIR) spectra of ground oyster shell granules in (**b**) indicate absorption peaks around 877 and 713 cm^−1^. These are due to vibrations of the carbon–oxygen double bond in the carbonate ion of calcite, confirming that the ground oyster shells are primarily composed of calcite. The cores in (**c, d**) received a 1-mm surface layers of (**c**) sterile carbonate free sand (Control) or (**d**) calcite (ground oyster shells; +Calcite).

To assess the response of our subtidal silt sediment, we submerged in each of two experimental units circulating natural seawater at in situ temperature (Table 1) 12 intact sediment cores (Fig. 1) and provided 12 h d^−1^ of photosynthetically active radiation to the sediment surface at a flux similar to that measured midday at the 10 m-deep core collection site. We then deposited a 1 mm layer of < 125 μm sterile sand particles (six cores, Control, Fig. 1c) or ground oyster shell (six cores,  +Calcite, Fig. 1d) onto the sediment surface in each unit. Starting on d 2 of the 21-d experiment (Fig. S1), we gradually (0.04 pH units d^−1^) increased the seawater [H^+^] in one of the experimental units using CO_2_-enriched air from pH 8.0 (*p*CO_2_ = 472 μatm) to 7.6 (*p*CO_2_ = 1216 μatm, Table 1) and then, starting on d 16, measured vertical microprofiles of pH and [O_2_] under conditions of light and darkness in individual cores (Fig. S1).

**Table 1:** Properties of ambient and CO_2_ enriched seawater (mean ± 1 SD) in the experimental units.

	Ambient	CO_2_ enriched
**Measured parameters**		
Temperature	14.6 ± 0.15	14.7 ± 0.16
Salinity	34.5 ± 0.07	34.5 ± 0.06
DIC	2112 ± 5	2261 ± 13
TA	2302 ± 10	2314 ± 10
**Calculated parameters at 15 °C**		
pH_T_	7.99 ± 0.01	7.62 ± 0.03
[H^+^]	10.3 ± 0.2	23.6 ± 0.9
*p*CO_2_	472 ± 7	1216 ± 88
[CO_3_^2−^]	139 ± 3	66 ± 4
Ω_CA_	3.32 ± 0.06	1.57 ± 0.09
Ω_AR_	2.13 ± 0.04	1.01 ± 0.06

Temperature (°C) and salinity are averages of 22 daily measurements whereas DIC and TA (µmol kg SW^−1^) are averages of five measurements taken during the final 10 d of the experiment when the pH was stabilised at the target value (Fig. S1). Seawater pH (total scale), [H^+^] (nmol L^−1^), *p*CO_2_ (μatm), [CO_3_^2−^] (µmol kg SW^−1^). The calcite and aragonite saturation states (Ω_CA_ and Ω_AR_, respectively) were derived from the measured parameters for a temperature of 15 °C.

## Results

In the following, we report H^+^/O_2_ concentrations ([H^+^], [O_2_]) and fluxes in the porewater of Control sediment under conditions of light and darkness, first in ambient seawater and then in CO_2_-enriched seawater. Following this, we assess how the addition of a surface calcite layer (+Calcite) altered porewater [H^+^] and [O_2_], again, first in ambient seawater and then in CO_2_-enriched seawater.

Our pH microprofiles describe depth gradients in proton concentrations [H^+^] ([H^+^] = 10^−pH^). Because the range of [H^+^] observed in this experiment was small, we do not report these as pH but instead as nmol L^−1^. We note that within the range of pH measured in our experiment, the [H^+^] is directly proportional to the [CO_2_] because CO_2_ and H^+^ have a 1:1 stoichiometry (CO_2_ + H_2_O <-> H_2_CO_3_ <-> H^+^ + HCO_3_^−^). The equilibrium activity ratio of CO_2_ to HCO_3_^−^ is presented in Fig. S3.

### Control: diel oscillations in porewater [H^+^] and [O_2_] in ambient seawater

**Figure 2:**
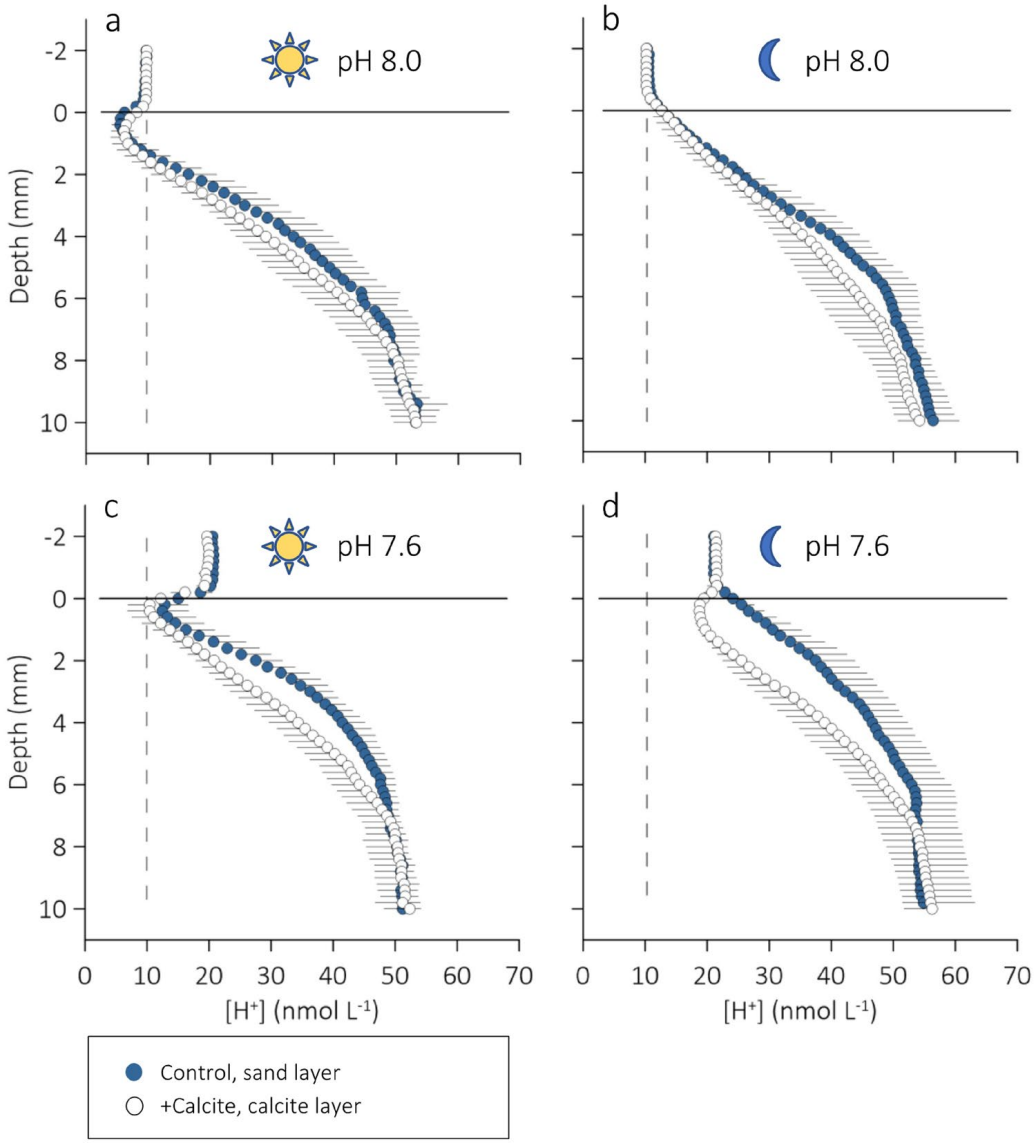
Average (n = 5) vertical microprofiles of porewater [H^+^] measured in intact cores of subtidal silt submerged in (**a**, **b**) ambient seawater and (**c**, **d**) CO_2_-enriched seawater under conditions of (**a**, **c**) light and (**b**, **d**) darkness. A 1 mm surface layer of either sterile sand (blue symbols, Control) or calcite (open symbols, +Calcite) was added to the sediment cores at the start of the experiment. The dashed and solid lines indicate the [H^+^] in ambient seawater and the position of the sediment surface, respectively. Black horizontal lines extending to the right or left of the symbols indicate 1 SD. For equilibrium activity ratios of CO_2_ to HCO_3_^−^, see Figure S3.

**Figure 3:**
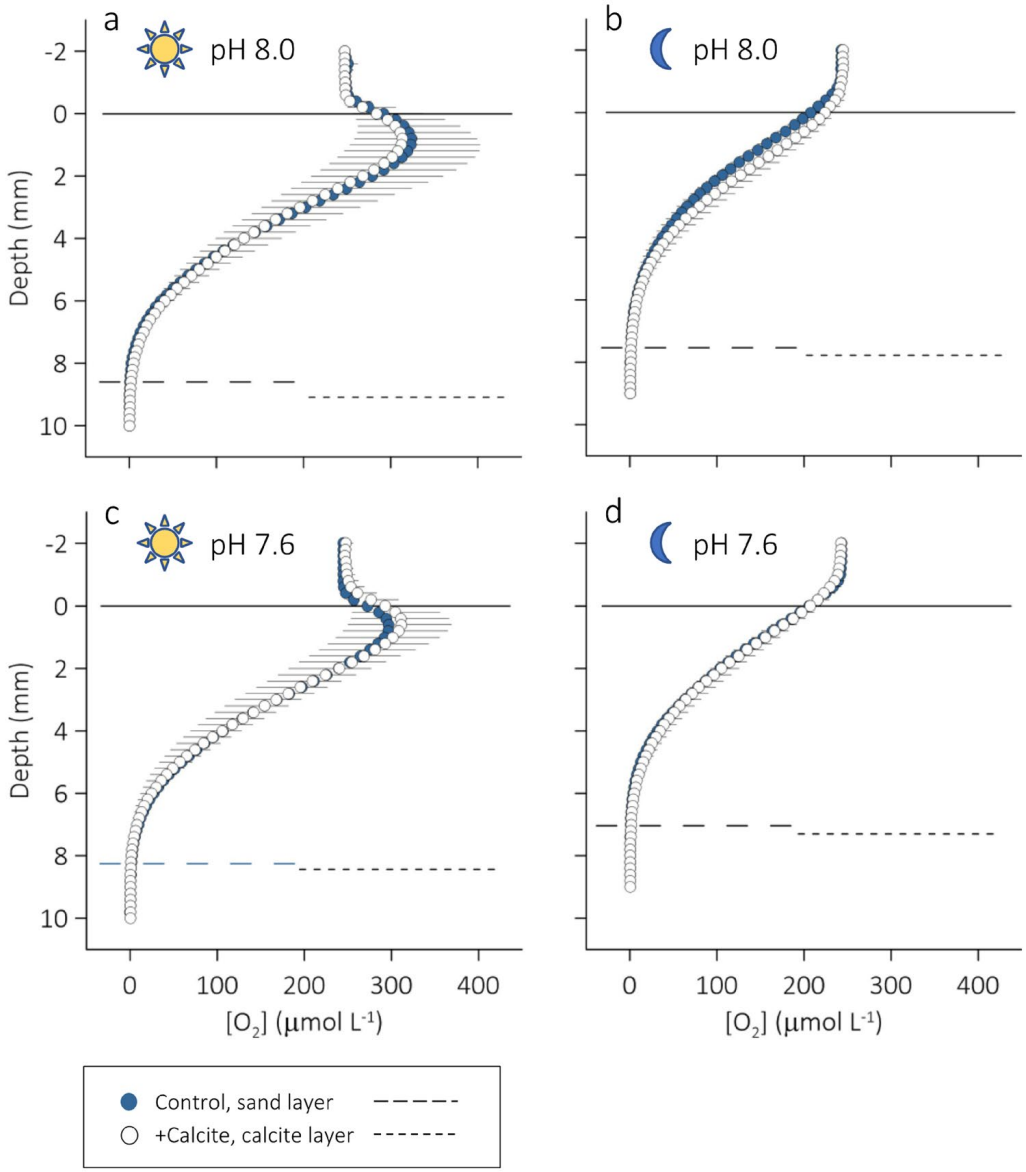
Average (n = 6) vertical microprofiles of porewater [O_2_] measured in intact cores of subtidal silt submerged in (**a**, **b**) ambient seawater and (**c**, **d**) CO_2_-enriched seawater under conditions of (**a**, **c**) light and (**b**, **d**) darkness. A 1 mm surface layer of either sterile sand (blue symbols, Control) or calcite (open symbols, +Calcite) was added to the sediment cores at the start of the experiment. The horizontal lines at 0 mm depth indicate the position of the sediment surface. Black horizontal lines extending to the right or left of the symbols indicate 1 SD. The dashed horizontal line indicates the average O_2_ penetration depth in Control (long dash) and +Calcite (short dash).

**Figure 4:**
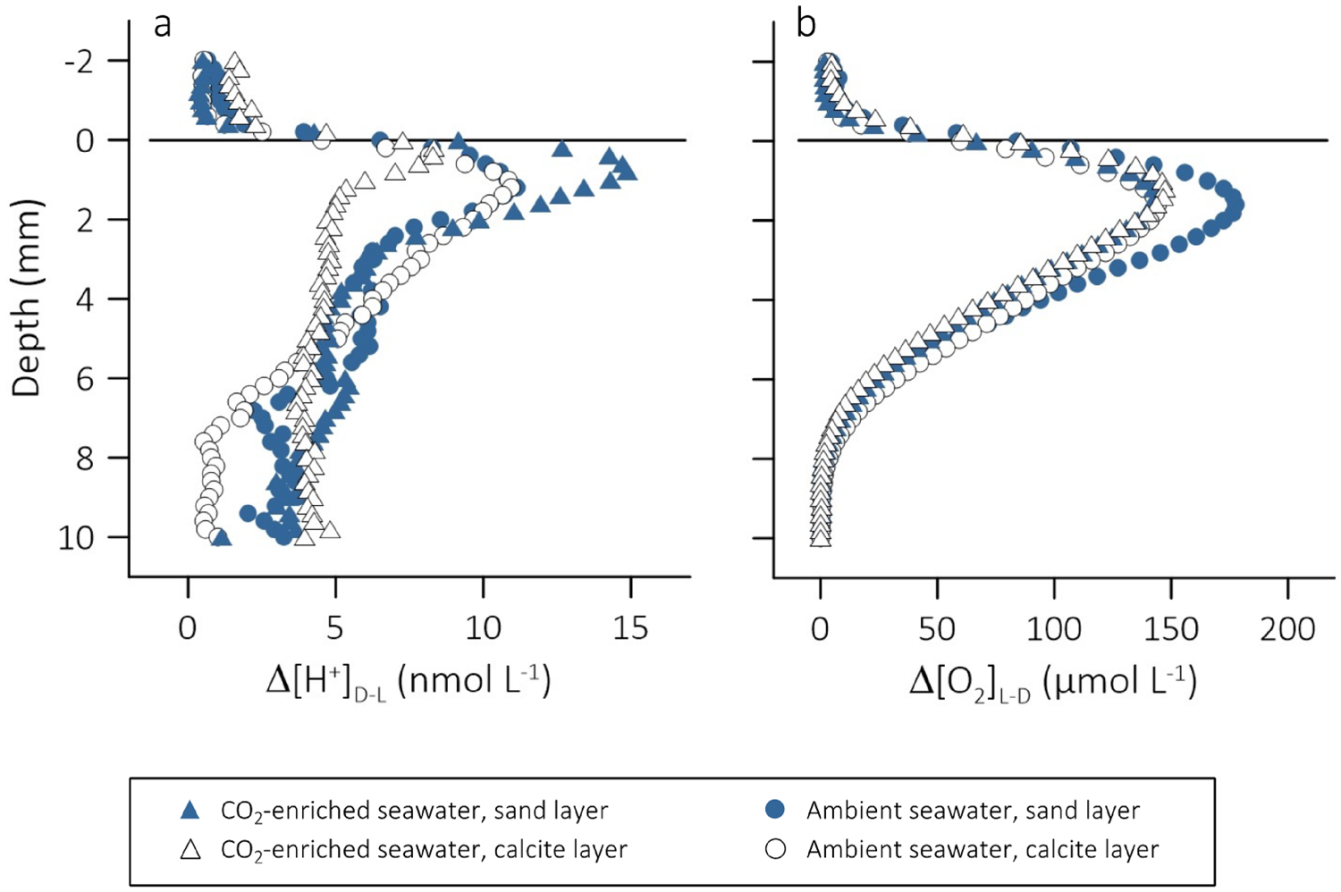
Diel oscillations in porewater (**a**) [H^+^] (Δ[H^+^]_D-L_, nmol L^−1^) and (**b**) [O_2_] (Δ[O_2_]_L-D_, µmol L^−1^) as a function of depth in the surface layer of intact cores of subtidal silt sediment submerged in ambient (circles) and CO_2_-enriched (triangles) seawater. A 1 mm surface layer of either sterile sand (filled symbols, Control) or calcite (open symbols, +Calcite) was added to the sediment cores. The horizontal line at 0 mm depth indicates the position of the sediment surface.

Porewater microprofiles measured in the Control sediment revealed that the photosynthetically induced diel oscillations in porewater [H^+^] and [O_2_] extended to depths of about 8 mm (Figs. 2, 3, 4). Integrated over the experimental 12/12 h light–dark cycle, the subtidal sediment was a sink for H^+^ and a source of O_2_. The [H^+^] gradients in the diffusive boundary layer (DBL) of the sediment—a thin (< 1 mm) film of water that covers the sediment, and through which molecular diffusion is the dominant transport mechanism for solutes—indicated that, on average, the sediment removed 2.5 times more H^+^ from the overlying seawater in light than it released in darkness (Table 2, H^+^ flux_DBL_).

**Table 2:** Effects of seawater CO_2_ enrichment and calcite deposit on the silt’s average (± 1 SD) O_2_ consumption, porewater oxygenation, and H^+^ flux.

	Control (+Sand)				+Calcite			
	pH 8.0	pH 7.6	*d* ES	CI	pH 8.0	pH 7.6	*d* ES	CI
**Light**	(6)	(6)			(6)	(6)		
O_2_-pd	8.5 ± 0.2	8.2 ± 0.7	0.56	0.28/0.83	9.0 ± 0.4	8.4 ± 0.4	1.56	1.36/1.75
DOE	489 ± 442	382 ± 277	0.32	− 190/191	478 ± 342	490 ± 337	− 0.04	− 175/175
*R*_A_	332 ± 247	315 ± 290	0.07	− 143/143	302 ± 198	377 ± 333	− 0.30	− 142/141
	(5)	(5)			(5)	(6)		
H^+^ flux_DBL_	− 217 ± 55	− 437 ± 220	1.53	− 87.3/90.4	− 156 ± 92	− 421 ± 140	2.41	− 62.5/67.3
H^+^ flux_sub_	96 ± 16	42 ± 19	3.42	− 6.4/13.2	121 ± 17	84 ± 3	3.51	− 2.78/9.79
**Darkness**	(6)	(5)			(6)	(6)		
O_2_-pd	7.6 ± 0.5	7.0 ± 0.7	1.32	1.04/1.60	7.8 ± 0.6	7.3 ± 0.6	0.97	0.66/1.28
DOE	− 263 ± 52	− 293 ± 55	0.60	− 28.0/29.2	− 178 ± 64	− 239 ± 33	1.31	− 25.1/27.7
*R*_A_	− 229 ± 14	− 254 ± 53	0.73	− 19.0/20.4	− 219 ± 38	− 235 ± 36	0.47	− 18.5/19.4
*R*_V_	− 30 ± 2.2	− 37 ± 9.6	1.20	− 2.32/4.72	− 28 ± 7.0	− 32 ± 6.9	0.65	− 2.93/4.23
	(4)	(4)			(6)	(6)		
H^+^ flux_DBL_	87 ± 46	152 ± 92	− 0.97	− 47.0/45.0	106 ± 90	− 155 ± 111	2.83	− 49.4/55.1
H^+^ flux_sub_	55 ± 21	41 ± 12	0.94	− 9.2/11.1	84 ± 20	95 ± 33	− 0.44	− 14.6/13.7

O_2_-pd, O_2_ penetration depth (mm); DOE, diffusive O_2_ exchange (μmol m^−2^ h^−1^); *R*_A_, depth-integrated O_2_ production (μmol m^−2^ h^−1^); *R*_V_, integrated volume-specific O_2_ production (= *R*_A_/O_2_-pd, nmol cm^−3^ h^−1^). H^+^ flux_DBL_ and H^+^ flux_sub_ (mmol m^−2^ h^−1^), diffusive H^+^ flux calculated from the vertical [H^+^] gradients in the diffusive boundary layer and at the bottom 2 mm of the oxic layer, respectively. Negative and positive DOE, *R* or H^+^ flux values indicate sediment solute uptake and release, respectively. The *d* Effect size (*d* ES) and 95% confidence intervals (CI) refer to pH 8.0 (ambient) versus pH 7.6 (CO_2_-enriched) comparisons. Numbers in parenthesis are number of replicate measurements (cores).

Visual inspection of the sediment surfaces revealed that the motile pennate diatoms species that dominated the microphytobenthos of this sediment (see Vopel et al. 2018; genera *Pleurosigma*, *Gyrosigma*, *Nitzschia*, *Thalassionema*, and *Bacillaria*) relocated to the surface within 3 h of the sand and calcite layers being added (Fig. 1a). Photosynthesis lowered the [H^+^] in the top millimetre of the sediment below that measured in the overlying seawater (Fig. 2a). This reversed the direction of the sediment–seawater H^+^ exchange observed in darkness and increased the flux of H^+^ from the bottom of the oxic zone to the surface sediment by a factor of 1.7 (H^+^ flux_sub_ in Table 2).

Vertical [O_2_] microprofiles measured in the DBL to compute the diffusive sediment–seawater O_2_ exchange (DOE), and in the sediment porewater to compute the depth-integrated sediment O_2_ production (*R*_A_) showed that the Control released on average 1.9 and 1.4 times, respectively, more O_2_ in light than it consumed O_2_ in darkness (Table 2). Note the large standard deviation of the average DOE measured in light indicating patchiness in the distribution of benthic microphytes (Fig. 1a). In darkness, O_2_ diffused from the freely flowing seawater above the sediment to an average sediment depth of 7.6 mm (Fig. 3b). As expected, photosynthesis at the surface of the sediment supersaturated the porewater of the top 2 mm of the sediment with O_2_, reversing the dark O_2_ flux and increasing the O_2_ penetration by 0.9 mm (Fig. 3a). Note that when all the derived DOE and *R*_A_ data (Table 2) were pooled, they were linearly correlated (R^2^ = 0.96), but agreement between these two estimates gradually decreased with increasing O_2_ production (Fig. S2).

### Control: effects of seawater CO_2_ enrichment

In light, the Control sediment removed H^+^ from the overlying CO_2_-enriched seawater (H^+^ flux_DBL_) at a larger rate than from the ambient seawater (Tables 2, 3; Fig. 2a,c). As expected, a greater H^+^ uptake from the CO_2_-enriched seawater was accompanied with a smaller flux from the bottom of the oxic layer (H^+^ flux_sub_, Tables 2, 3). Note that the increase in H^+^ uptake from the CO_2_-enriched seawater outweighed the decrease in flux from the bottom of the oxic sediment layer. That is, in light, the surface layers of the Control sediment consumed more H^+^ in CO_2_-enriched seawater than in ambient seawater. In darkness, the flux of H^+^ across the DBL of was independent of the seawater *p*CO_2_ (Tables 2, 3; Fig. 2b,d).

**Table 3:** Summary of two-way ANOVA *p* values in light and darkness testing for the effects of seawater *p*CO_2_ (ambient (amb), enriched (enr)) and sediment surface deposit (sand (san), calcite (cal)) on the silt’s O_2_ penetration depth (O_2_-pd), diffusive O_2_ exchange (DOE), depth integrated O_2_ consumption (*R*_A_), depth integrated volume specific O_2_ consumption (*R*_V_) H^+^ fluxes across the diffusive boundary layer (H^+^ flux_DBL_) and at the bottom of the oxic sediment layer (H^+^ flux_sub_).

	*p*CO_2_	Deposit	*p*CO_2_ × deposit	Tukey post-hoc test results
**(A) Light**				
O_2_-pd	0.0428	0.2724	0.7552	
DOE	0.8116	0.7366	0.9599	
*R*_*A*_	0.9551	0.7946	0.7127	
H^+^ flux_DBL_	0.0128	0.7591	0.2052	
H^+^ flux_sub_	0.0029	< 0.0001	0.1100	
**(B) Darkness**				
O_2_-pd	0.0235	0.3716	0.8350	
DOE	0.0532	0.0048	0.4776	
*R*_*A*_	0.2498	0.4024	0.8026	
*R*_*V*_	0.0883	0.3647	0.6326	
H^+^ flux_DBL_	0.0335	0.0036	0.0014	amb: san versus cal, *p* = 0.9887; enr: san versus cal, *p* = 0.0007 san: amb versus enr, *p* = 0.7611; cal: amb versus enr, *p* = 0.0010
H^+^ flux_sub_	0.8980	0.0017	0.2787	

Seawater CO_2_ enrichment also affected the O_2_ penetration depth (O_2_-pd) in darkness; O_2_ penetrated the Control less from the CO_2_-enriched seawater than from the ambient seawater (Tables 2, 3). The effects of seawater CO_2_ enrichment on the volume-specific sediment O_2_ production, *R*_V_, and the DOE, however, were statistically not clear.

### +Calcite: effects of calcite deposition in ambient seawater

In light, the flux of H^+^ from the bottom of the oxic sediment layer (H^+^ flux_sub_) into the calcite surface layer exceeded that into the sand surface layer (Control) by a factor of 1.3 (Tables 2, 3; Fig. 2a,c). Similarly, in darkness, the flux of H^+^ from the bottom of the oxic sediment layer of the +Calcite treatment exceeded that from the bottom of the oxic layer of the Control by a factor of 1.5 (Tables 2, 3; Fig. 2b,c). The calcite surface layer had no statistically clear effect on the H^+^ flux across the DBL (H^+^ flux_DBL_, Table 2).

In light, Control and +Calcite did not differ in any of the measured or derived sediment O_2_ consumption proxies (Tables 2, 3, Fig. 3a). In darkness, the +Calcite cores removed on average less O_2_ from the overlying seawater than the Control cores (DOE in Tables 2, 3, Fig. 3b). However, the estimates of *R*_A_ and the volume-specific O_2_ production, *R*_V_, did not confirm this difference (Tables 2, 3).

### +Calcite: effects of calcite deposition in CO_2_ enriched seawater

The effects of calcite deposition on the light and dark fluxes of H^+^ from the bottom of oxic layer was greater in CO_2_-enriched seawater than in ambient seawater: H^+^ flux_sub_ into the calcite surface layer (+Calcite) exceeded that into the sand surface layer (Control) by factors of 2.0 and 2.3 in light and darkness, respectively (Tables 2, 3; Fig. 2c,d).

In light, calcite deposition had no statistically clear effect on H^+^ flux_DBL_ (Table 2, Fig. 2c). In darkness, however, this resulted in a H^+^ flux_DBL_ similar in size but opposite in direction of that in the Control (152 ± 98 vs. − 155 ± 111 mmol m^−2^ h^−1^, Table 2, Fig. 2d). The effect of calcite deposition on the the measured or derived sediment O_2_ consumption proxies was statistically not clear, in both light and darkness (Tables 2, 3).

## Discussion

**Figure 5:**
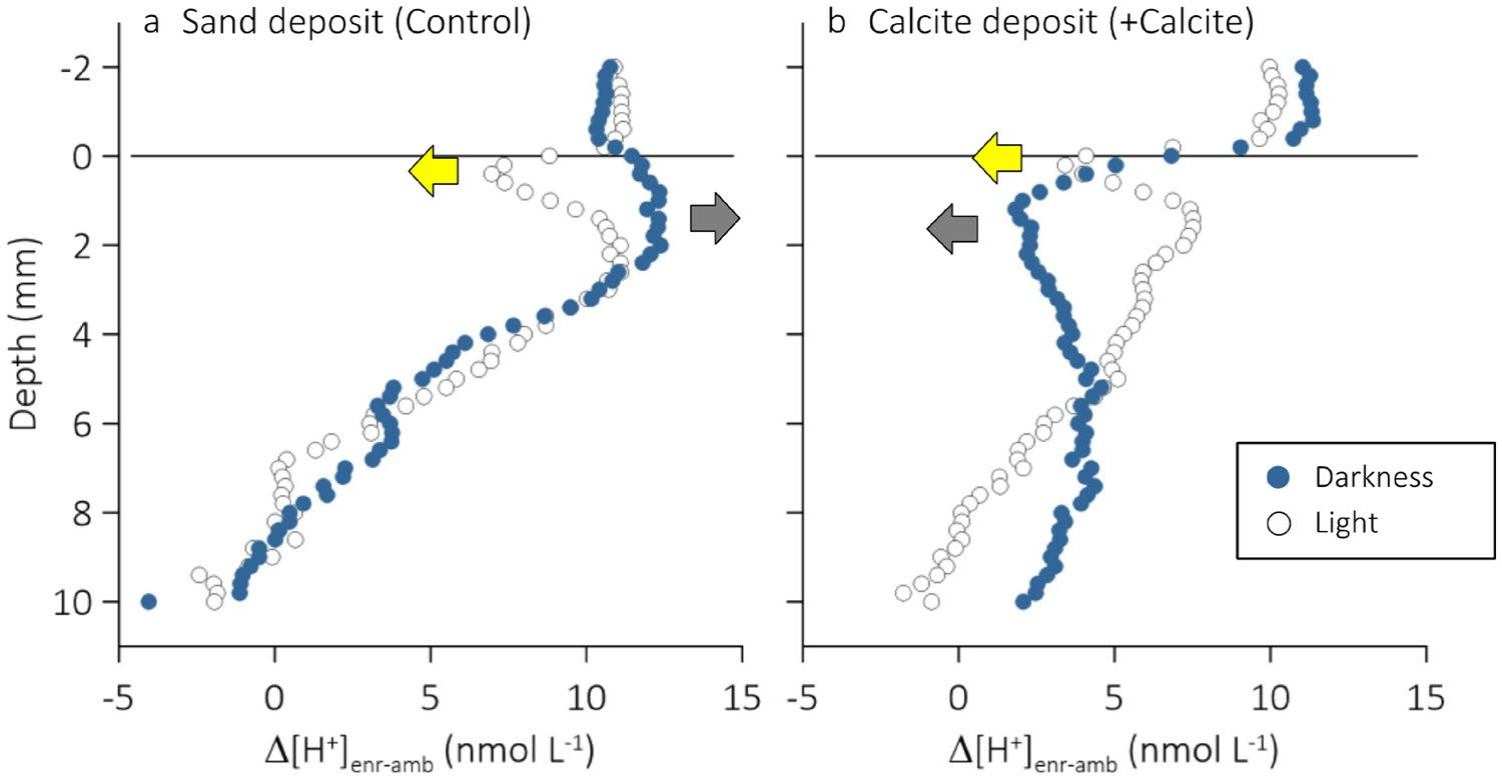
Effect of seawater CO_2_ enrichment on the sediment porewater [H^+^] (Δ[H^+^]_enr-amb_, nmol L^−1^) under conditions of light (open symbols; yellow arrow) and darkness (closed symbols; shaded arrow) and added (**a**) sterile sand (Control) or (**b**) calcite (+Calcite).

Our measurements in cores of a photosynthetic subtidal silt sediment with 1 mm of added sterile sand (Control) revealed that enrichment of the overlying seawater with CO_2_ had increased the peak-to-peak amplitude of the diel porewater [H^+^] oscillations (Fig. 4a, compare filled triangles vs. filled circles). Two effects likely contributed to this increase: (1) in darkness, sedimentary microbial reaction processes seem to have amplified the positive effect of seawater CO_2_ enrichment on porewater [H^+^] (Fig. 5a, filled symbols), and (2) replenishment of the CO_2_ taken up during diatom photosynthesis by the bicarbonate pool may have consumed H^+^ at a greater rate in CO_2_-enriched seawater than in ambient seawater (Fig. 5a, open symbols). Furthermore, we showed that seawater CO_2_ enrichment decreased the sediment penetration of O_2_ in both light and darkness (Tables 2, 3) suggesting a CO_2_ response of microbial reaction processes that either directly or indirectly consumed O_2_.

The addition of biogenic calcite to the surface of the sediment increased the flux of H^+^ from the bottom of the oxic sediment layer (H^+^ flux_sub_) towards the sediment surface, in light and darkness, and in both ambient and CO_2_-enriched seawater (Tables 2, 3). We suspect that this was caused by dissolution of calcite at the sediment-facing boundary of the calcite deposit. The H^+^ flux across the seawater-facing boundary of the calcite deposit (H^+^ flux_DBL_), on the other hand, did not statistically differ from the flux across the seawater-facing boundary of the sand deposit, except in CO_2_-enriched seawater under conditions of darkness (Tables 2, 3). Under these conditions, seawater CO_2_ enrichment raised the porewater [H^+^] sufficiently to trigger calcite dissolution across the entire deposit. This dissolution then decreased the porewater [H^+^] below that in the CO_2_-enriched overlying seawater reversing the direction of the H^+^ flux (Fig. 2d). Consequently, the peak-to-peak amplitude of the diel porewater [H^+^] oscillations decreased (Fig. 4a, open triangles). Such an effect was not observed in ambient seawater: the overlapping Δ[H^+^]_D-L_ profiles in Fig. 4a (open and closed circles) indicate that in ambient seawater, the influences of photosynthesis on [H^+^] in the porewater of Control and +Calcite were similar. That is, in ambient seawater, the addition of calcite did not alter the photosynthetically induced [H^+^] oscillations.

Besides differences between Control and +Calcite in the flux of H^+^ from the bottom of the oxic sediment layer, the O_2_ gradients in the diffusive boundary layer (DBL) measured in darkness under conditions of ambient and CO_2_-enriched seawater revealed differences between Control and +Calcite in the diffusive O_2_ exchange (DOE, Tables 2, 3). These gradients may not correctly reflect the steady-state sediment–seawater O_2_ exchange, but if so, then the +Calcite cores seemed to have removed less O_2_ from their overlying seawater than the Control cores in both ambient (*d* ES = − 1.6) and CO_2_-enriched seawater (*d* ES =  − 1.35). We note, however, that the proxies derived from the measured porewater [O_2_] profiles (O_2_-pd, *R*_A_ and *R*_V_) did not return statistically clear differences.

We hypothesise that the observed increase in the flux of H^+^ from the bottom of the oxic layer of the +Calcite sediment resulted from the dissolution of the sediment-facing boundary of the calcite deposit. Inspection of the [H^+^] microprofiles shown in Fig. 2b reveals that this may have started at a porewater [H^+^] of 15–20 nmol L^−1^ (pH 7.8–7.7)—the concentrations measured at 1 mm depth. We can infer from the Control that CO_2_ enrichment of the sediment-overlying seawater then raised the [H^+^] in the porewater of the calcite layer above ~ 25 nmol L^−1^ (below pH 7.6, Fig. 2d, Control) initiating dissolution, which lowered the porewater [H^+^] to about 20 nmol L^−1^ (increased pH to ~ 7.7) as shown in Fig. 2d. If the dissolution–precipitation balance was to shift toward net dissolution at Ω_CA_ < 1, and assuming that porewater total alkalinity, TA = 2.3 mmol kg^−1^, then calcite should be stable at pH > 7.4, and the [H^+^] profiles across the calcite layer should resemble the profiles measured in the Control. Our results suggest that dissolution started at a lower [H^+^] (higher pH) confirming the evidence presented by others^24^ who have observed that gross dissolution of whole-shell biogenic CaCO_3_ occurred in treatments that were oversaturated (Ω > 1) with respect to calcite.

The abundance of large motile pennate diatoms raises the possibility that the dissolution of the surface calcite was enhanced by the activity of extracellular enzymes. Diatoms use an extracellular carbonic anhydrase^25,26^, located in the periplasmatic space^27,28^, as part of a carbon-concentrating mechanism that increases the flux of CO_2_ towards the carboxylating enzyme, ribulose-1,5-bisphosphate carboxylase-oxygenase (RubisCO)^29^. This enzyme catalyses the otherwise slow inter-conversion of CO_2_ and HCO_3_^−^ and in this case, facilitates CO_2_ uptake by generating CO_2_ from HCO_3_^−^ at the cell surface^26^ through a two-step process, a hydration–dehydration step followed by a rate-limiting transfer of protons, which is presumably buffered by the acidic polymerised silica of the diatoms’ cell wall^30^. Evidence presented by Subhas et al.^31^ suggests that carbonic anhydrase increases calcite dissolution for saturation states from 0.6 to near 1, the effect being most pronounced close to equilibrium (Ω_CA_ = 1).

### What amplified the effects of biogeochemical processes on porewater [H^+^] in CO_2_-enriched seawater?

In Fig. 5, we use vertical Δ[H^+^]_enr-amb_ profiles to assess how seawater CO_2_ enrichment altered [H^+^] in both the sediment-overlying seawater and the sediment porewater. As mentioned above, for both Control and +Calcite, injection of CO_2_-enriched air will initially have decreased the porewater–seawater [H^+^] gradient observed in darkness and so the dark H^+^ efflux. If sedimentary reaction processes kept producing and consuming H^+^ at unchanged rates, then the porewater [H^+^] and sediment–seawater H^+^ efflux must have gradually increased until net production and efflux were again in equilibrium. That is, the [H^+^] at the sediment surface must have increased by as much as the [H^+^] in the bottom seawater and Δ[H^+^]_enr-amb_ would gradually be attenuated with sediment depth. However, Fig. 5a shows that in darkness, Δ[H^+^]_enr-amb_ in the porewater of the upper 2 mm of the sediment exceeded that in the overlying seawater. This suggests that net-production of H^+^ had increased leading to higher porewater concentrations and a higher H^+^ efflux.

Following Middelburg et al.^32^, the instantaneous effect of a biogeochemical process on [H^+^] is the product of the net charge exchanged during the process (Δcharge), the sensitivity factor of seawater (*δpH/δCBA*; *CBA* = charge balance alkalinity), and the process intensity (*I*_process_, mol m^−3^ s^−1^):

1 $$\Delta pH = \Delta {\text{charge}} \times \left( {\frac{\partial pH}{{\partial CBA}}} \right) \times I_{process}$$

Because the sensitivity factor and the net charge exchange are functions of pH, the influence of a biogeochemical process also depends on pH. In other words, the acidification of the sediment porewater can alter the positive or negative effect of biogeochemical reaction processes on porewater [H^+^] even if the intensity of the reaction process remains unchanged. For aerobic mineralisation and the reoxidation of reduced solutes with O_2_, this pH dependency sees the production of [H^+^] steeply increasing as porewater [H^+^] increases above 10 nmol L^−1^ (pH decreases below 8.0^33^). That is, an increase in porewater [H^+^] will have increased the production of H^+^ by porewater microbial reaction processes raising Δ[H^+^]_enr-amb_ above that observed in the overlying seawater, even if the reaction process intensity remained unchanged. It would follow then that the observed increase in Δ[H^+^]_enr-amb_ does not necessarily imply that an ecosystem process has responded to additional CO_2_.

While in darkness Δ[H^+^]_enr-amb_ in the porewater of the Control exceeded that in the overlying seawater, in light, it steeply decreased below that in the overlying seawater reaching a minimum just below the sediment surface (Fig. 5a). This suggests photosynthesis must have removed more H^+^ in CO_2_-enriched seawater than in ambient seawater. Again, this may have followed from the pH dependencies of the seawater sensitivity factor and the net charge exchange (see above); Soetaert et al.^33^ showed that the consumption of porewater H^+^ by photosynthesis based on ammonium or nitrate increases steeply as the environmental [H^+^] increases above 10 nmol L^−1^. An increase in the process intensity, that is, CO_2_-enhanced photosynthetic carbon fixation, on the other hand, should be reflected in the measured [O_2_] microprofiles showing an increase in O_2_-pd and sediment–seawater O_2_ release. There is, however, no evidence for such an effect (Figs. 3b, 4a,c, Table 2). None of the [O_2_] microprofiles and O_2_ production estimates indicate a greater net production of O_2_ in CO_2_-enriched seawater.

Besides enhancing the H^+^ production (dark) and consumption (light) in the porewater of the surface sediment, seawater enrichment with CO_2_ caused a statistically clear decrease in O_2_-penetration depth (O_2_-pd, *d* ES 1.3), and, although statistically not clear, an increase in the average integrated volume-specific O_2_ consumption (negative *R*_V_, Control, *d* ES = 1.2). The latter effect and the elevated Δ[H^+^]_enr-amb_ may have a common cause, a positive CO_2_ response of microbial reaction processes that generate H^+^ *and* consume O_2_: the microbial reoxidation of reduced solutes (iron, manganese and sulphur) with O_2_ and aerobic mineralisation and nitrification. For example, an increase in the production and subsequent oxidation of Fe^2+^ would have raised both the O_2_ demand and the porewater [H^+^] in the surface sediment adding to the effects of the pH-dependent sensitivity factor and net exchange of charge, as per Eq. (2).

2 $$4{\text{Fe}}^{2 + } + {\text{HCO}}_{3}^{-} + 10{\text{H}}_{2} {\text{O}} \to 7{\text{H}}^{ + } + 4{\text{Fe}}\left( {{\text{OH}}} \right)_{3} + {\text{CH}}_{2} {\text{O}}$$

Here, for the first time, we presented evidence for a positive effect of seawater CO_2_ enrichment on the peak-to-peak amplitude of the dark–light oscillation in porewater [H^+^] that characterise cohesive photosynthetic sediment. We discussed possible causes of this effect and showed that dissolution of carbonate, if added to the surface, will diminish these oscillations. The pH dependencies of both the seawater sensitivity factor and the net exchange of charge may explain why sediment biogeochemical processes in CO_2_-enriched seawater added and removed more H^+^ to and from the porewater in darkness and light, respectively, than they did in ambient seawater. Ecosystem feedbacks in the form of CO_2_-induced changes in the intensity of photosynthesis in light and respiration in darkness may also explain an enhanced consumption and production, respectively, of porewater H^+^, but this is not consistent with the observed decrease in O_2_ penetration in light and the similarity of the [O_2_] microprofiles measured in ambient and CO_2_-enriched seawater. One possible process that explains this discrepancy is increased microbial reoxidation of reduced solutes with O_2_, and thus increased sediment O_2_ demand, decreasing the O_2_ penetration depth while increasing porewater [H^+^]. The dissolution of the added calcite then effectively countered the increase in porewater [H^+^] (Fig. 5b), but not the CO_2_-induced decrease in O_2_ penetration. This dissolution should not be considered an ecosystem feedback because calcite was not naturally present at the sediment surface. It indicates, however, a challenge for recruits of calcifying fauna arriving at the sediment surface. A chemically more aggressive (acidified) surface layer may prevent macrofauna recruitment^20–22^ and so alter the three-dimensional complexity of the sedimentary ecosystems^34^ and associated carbon and nutrient remineralisation and sediment–seawater solute exchange processes.

## Material and methods

### Sediment collection and properties

On June 11th 2019, we collected 24 cores of subtidal silt with SCUBA at 10 m water depth in Man O’War Bay (S 36° 47′ 38″, E 175° 10′ 14″), Hauraki Gulf, New Zealand, as described in Vopel et al.^19,35^. The cores were kept below 15 °C, the in situ seawater temperature, during a 2.5 h trip to the laboratory. The salinity of the seawater was 34.5. Loss of weight of the silt’s upper 10 mm layer after drying at 60 °C and combustions at 550 and 950 °C indicated that on average, porewater accounted for 68 ± 1.3% of the silt’s wet weight, and 9.5 ± 0.4 and 4.1 ± 0.2% of the silt’s dry weight were organic matter and the calcium carbonate, respectively (± 1 SD, n = 3). Chlorophyll *a* and phaeopigment contents determined following Jeffrey and Humphrey^36^ were 12.5 ± 1.9 and 20.5 ± 2.4 μg (g dry weight)^−1^ (n = 3) giving a Chl *a*/phaeopigment ratio of 0.6. Particle sizes, determined using ~ 10 mL sample of the homogenized silt and a Malvern Mastersizer 2000, ranged from 0.24 to 350 μm with a volume weighted mean of 35.7 ± 0.9 μm (Kurtosis: 8.0 ± 1.3, Skewness: 2.6 ± 0.2).

### Laboratory setup and seawater properties

We submerged 12 randomly selected sediment cores in two independent experimental units (EU) each circulating ~ 1120 L of natural seawater (Table 2). Details of these units are described in Vopel et al.^19,35^. LED floodlights provided ~ 130 μmol quanta m^−2^ s^−1^ of photosynthetically active radiation (PAR) from 7 a.m. to 7 p.m. at the surface of the submerged sediment cores. This intensity was similar to that measured midday at the core collection site (K. Vopel unpublished observations). The seawater in each circulation unit was continuously sterilised with UV light and a sprinkler returning seawater from an in-line particle filter ensured that the seawater was saturated with O_2_ (K. Vopel, unpublished observations).

Starting on d 2 of the experiment, we manipulated the seawater circulating in one EU by automatic stepwise injection of CO_2_-enriched air (5% CO_2_, 21% O_2_ in nitrogen) to decrease its pH by 0.04 units per day (for 10 d) until a pH of 7.60 was reached (Fig. S1). The seawater pH was then maintained at 7.60 for a further 9 d until the end of the experiment (d 21). The level of CO_2_ enrichment was controlled by CapCtr software (Loligo Systems Aps), a SenTix HWD electrode connected to a pH 3310 m (WTW), and a solenoid valve. For additional details including the calibration of pH electrodes see Vopel et al.^35^.

We measured the seawater salinity daily and kept it between 34.4 and 34.7 by adding ultrapure water to account for evaporation. Seawater analyses in previous experiments (K. Vopel unpublished observations) revealed that this addition had no measurable effect on the seawater carbonate chemistry. One litre of seawater collected from each EU weekly was analysed for dissolve inorganic carbon (DIC) with a SOMMA (Single Operator Multiparameter Metabolic Analyzer) coulometer system, and total alkalinity (TA) with a closed-cell potentiometric titration system following the SOP’s 2 and 3a procedures^37^. D. Pierrot’s adaptation of the CO_2_Sys.BAS program^38^ computed the seawater *p*CO_2_ and pH (total scale, mol kg-SW^−1^). The dissociation constant for HSO_4_^−^ was taken from Dickson^39^; the values of K_1_ and K_2_ of carbonic acid were from Mehrbach et al.^40^ refitted by Dickson and Millero^41^. The CO_2_Sys.BAS computations confirmed that the stepwise increase in the injection of CO_2_-enriched air increased the seawater *p*CO_2_ by a factor of ~ 2.6 and decreased seawater pH from 8.0 to 7.6 (Table 1).

### Sediment treatment

On d 1 of the experiment, we added a 1 mm thick surface layer of biogenic calcite (Fig. 1) to six cores in each of the two experimental units. To do so, we briefly ground (Omni Ruptor 4000 Ultrasonic Homogenizer) bleach sterilised and rinsed oyster shells and sieved the material to exclude particles > 125 µm. Fourier transform infrared spectroscopy of this material confirmed the identity of the CaCO_3_ mineral (Fig. 1). We weighed 2 g into each of 12 seawater-filled 100-mm diameter petri-dishes to create a consistent, 1 mm thick layer at the bottom of each petri dish. The content of each petri dish was then frozen at − 80 °C and one of the resulting solid seawater/calcite disks was placed into the headwater space of each core. As the disks thawed, an even layer of calcite was distributed onto the surfaces of the sediment cores. To avoid disturbing the settling particles, the cores were isolated from the surrounding seawater until the surface layers of calcite had formed (~ 20 min). A 1 mm thick surface layer of sand particles was added to each of the remaining cores as a control using the same technique. The sand particles were first heated for 4 h at 550 °C to remove organic carbon and then sieved to exclude particles > 125 µm. After sieving, they were heated for a second time for 1 h at 840 °C to remove CaCO_3_.

### O_2_ and pH microprofiling and profile analyses

Starting on d 16 (i.e. after 4 d at pH = 7.6 in the enriched treatments, Fig. S1) of the experiment, we recorded one vertical [O_2_] and pH microprofile in each core under conditions of darkness and light. Measurements were made after at least 6 h exposure to light or darkness and the cores were selected at random across the 5 d it took to complete all the measurements. The vertical profiles were made at a 0.2 mm resolution starting from a position 2 mm above the sediment surface into the anoxic sediment at 9–10 mm depth with PreSens (optodes) and Unisense (pH microelectrodes, 100-µm tip) hard- and software.

We used the linear [O_2_] or [H^+^] gradients in the DBL of the sediment to calculate the diffusive O_2_ (DOE, µmol m^−2^ h^−1^) or H^+^ (H^+^ flux_DBL_, mmol m^−2^ h^−1^) sediment–seawater fluxes, respectively. To derive the flux of H^+^ from the deeper sediment (H^+^ flux_sub_, mmol m^−2^ h^−1^), we used the slope of the [H^+^] profile in the bottom 2 mm of the oxic layer. The O_2_ penetration depth (O_2_-pd, mm) was defined as the depth at which the porewater [O_2_] decreased below 1 µmol L^−1^. The sediment section of the [O_2_] profiles was used to derive the silt’s areal, depth integrated O_2_ consumption (*R*_A_, µmol m^−2^ h^−1^), and integrated volume-specific O_2_ production (*R*_V_ = *R*_A_/O_2_-pd, nmol cm^−3^ h^−1^), with the model PROFILE^42^ neglecting the sediment biodiffusivity and irrigation. Estimates of the porosity of the top 10 mm sediment, based on microelectrode measurements of apparent gas diffusivity in intact sediment cores (for technical details, see Revsbech et al.^43^ and Vopel et al.^44^), revealed an average value of *ϕ* = 0.85 (data not shown). The molecular diffusion coefficients of O_2_ and H^+^ (*D*_0_) were from Broecker and Peng^45^ and Cussler^46^, respectively, and corrected for temperature and salinity as described by Li and Gregory^47^. The diffusivities corrected for tortuosity were calculated as *D*_S_ = *D*_0_ × *ϕ*, following Ullman and Aller^48^. The difference in molecular diffusivity between the silt and the overlying seawater caused a distinct change in the slope of the measured [O_2_] profile at a position that marked the silt surface^49,50^.

Carbonate species distributions were determined in PHREEQC 3.0 using the seawater composition presented in Nordstom et al.^51^ to calculate the activity of associated inorganic complexes. Calculations were completed for each microprofile depth. While the total alkalinity in pore waters was not known, the activity ratio of CO_2_:HCO_3_ is independent of [DIC] and can be computed using the relevant equilibrium constants and the concentration of protons in solution.

### Statistical data analyses

The vertical profiles of Δ[H^+^]_D-L_ and Δ[H^+^]_enr-amb_ shown in Figs. 4 and 5 were calculated using averages of [H^+^] measured in replicate cores under conditions of light (L) and darkness (D) or in acidified (enr) and ambient (amb) seawater, respectively. We used the language proposed by Dushoff et al.^52^ when describing the conclusion from our statistical tests in terms of statistical ‘clarity’ rather than ‘significance’. (These authors argue against the over-reliance on a single (somewhat arbitrary) *p* value for determining the ‘significance’ of a test result). We used a two way analysis of variance (ANOVA) to test the effects of seawater *p*CO_2_ (ambient, enriched) and sediment surface deposit (sand, calcite) and their interaction on O_2_-pd, DOE, *R*_A_, H^+^ flux_DBL_ and H^+^ flux_sub_ under light and dark conditions. When a clear *p*CO_2_ × Deposit interaction was detected, separate Tukey HSD post-hoc analyses were undertaken for Deposit under *p*CO_2_ conditions and vice versa. All statistical tests were conducted using Statistica (StatSoft GmbH). Prior to analysis, data were checked for normality and homogeneity of variance (visual inspection of residuals); no transformations were required. The *d* Effect Size (*d* ES) and 95% Confidence Interval were computed using the Excel routine created by Jared DeFife, Emory University, 2009 (http://web.cs.dal.ca/~anwar/ds/Excel4.xlsx).

## Supplementary Information

Supplementary Information.

## Data Availability

The datasets are available from the corresponding author on reasonable request.
