# Supplementary material for: Biogeochemical feedbacks to ocean acidification in a cohesive photosynthetic sediment
Source: Sci Rep. 2021 Nov 24;11:22867. doi: 10.1038/s41598-021-02314-y (PMC8613249; doi:10.1038/s41598-021-02314-y)
Supplement: Supplementary file 1 — Supplementary Information. [file 41598_2021_2314_MOESM1_ESM.docx]

# Supplementary material

Biogeochemical feedbacks to ocean acidification in a cohesive photosynthetic sediment

Kay Vopel, Alexis Marshall, Shelly Brandt, Adam Hartland, Charles Lee, Craig Cary, Conrad A. Pilditch

**Table S1. Sediment properties**. Average (± 1 SD, n = 4) properties of the upper 10 mm layer of silt cores modified by addition of a 1-mm layer of sand (Control) or calcite (+Calcite). These cores were submerged in ambient or CO_2_ enriched seawater.

|  | Ambient seawater | | CO_2_ enriched seawater | |
| --- | --- | --- | --- | --- |
|  | Sand | Calcite | Sand | Calcite |
| Silt/clay content (% volume <63 μm) | 74.4 ± 4.5 | 80.7 ± 1.1 | 77.0 ± 1.0 | 80.3 ± 1.9 |
| Median grain size (μm) | 26.7 ± 4.4 | 22.5 ± 1.5 | 24.8 ± 1.3 | 22.8 ± 2.8 |
| Water content (% dry weight) | 67.2 ± 1.7 | 68.0 ± 0.6 | 66.2 ± 1.9 | 67.1 ± 2.1 |
| Organic matter content (% dry weight) | 8.4 ± 0.2 | 10.4 ± 0.0 | 8.6 ± 0.1 | 10.0 ± 0.1 |
| CaCO_3_ content (% dry weight) | 3.8 ± 0.3 | 5.0 ± 0.4 | 3.8 ± 0.2 | 4.6 ± 0.2 |
| Chl *a* (μg [g dw]^-1^) | 11.3 ± 1.0 | 14.4 ± 4.6 | 15.0 ± 4.1 | 15.1 ± 2.6 |
| Phaeopigments (μg [g dw]^-1^) | 18.1 ± 1.9 | 21.2 ± 0.6 | 18.5 ± 1.8 | 19.4 ± 2.7 |
| Ratio Chl *a*/phaeopigments | 0.6 ± 0.1 | 0.7 ± 0.2 | 0.8 ± 0.2 | 0.8 ± 0.1 |

**Table S2.** Summary of two-way ANOVA *p* values testing for the effects of seawater *p*CO_2_ (ambient, enriched) and sediment surface deposit (sand, calcite) on photosynthetic pigments.

|  | *p*CO_2_ | Deposit | *p*CO_2_ × Deposit |
| --- | --- | --- | --- |
| Chl *a* | 0.21 | 0.36 | 0.39 |
| Phaeopigments | 0.52 | 0.06 | 0.29 |
| Chl *a*/phaeopigments | 0.07 | 0.87 | 0.62 |

**Figure S1** Experimental timeline (I-IV) and time series of seawater (**a**) *p*CO_2_ and [H^+^] and (**b**) dissolved inorganic carbon concentration (DIC) and total alkalinity (TA) in two recirculating experimental units. The seawater in one unit (open symbols) was gradually enriched with CO_2_. Arrows: I, application of sand (Control) and calcite (+Calcite) deposits to cores; II, injection of CO_2_ started to stepwise increase the seawater *p*CO_2_ in one experimental unit; III, CO_2_ injection maintains *p*CO_2_ at 1216 ± 88 μatm; IV, microprofiling measurements start. See text for further details.

**Figure S2** Scatter plot showing the linear relationship between the diffusive O_2_ exchange (DOE) across the diffusive boundary layer (DBL), and the areal depth-integrated O_2_ consumption (*R*_A_). DOE was derived from [O_2_] microprofiles in the DBL, whereas *R_A_* from porewater [O_2_] microprofiles. The solid and dashed lines indicate respectively the linear fit (R^2^ = 0.96) and 95% confidence intervals.

**Figure S3** Average (n = 5) vertical microprofiles of porewater CO_2_:HCO_3_^–^ activity ratios measured in intact cores of subtidal silt submerged in (**a**, **b**) ambient seawater and (**c**, **d**) CO_2_-enriched seawater under conditions of (**a**, **c**) light and (**b**, **d**) darkness. A 1 mm surface layer of either sterile sand (blue symbols) or calcite (open symbols) was added to the sediment cores at the start of the experiment. The horizontal solid lines indicate the position of the sediment surface. The CO_2_:HCO_3_^–^ activity ratios were calculated in PHREEQC 3.0.
